# Improving hand hygiene measures in low-resourced intensive care units: experience at the Kigali University Teaching Hospital in Rwanda

**DOI:** 10.3396/ijic.v17.20585

**Published:** 2021-09-10

**Authors:** Jean Paul Mvukiyehe, Eugene Tuyishime, Anne Ndindwanimana, Jennifer Rickard, Olivier Manzi, Gregory R. Madden, Marcel E. Durieux, Paulin R. Banguti

**Affiliations:** 1 Department of Anesthesia, Critical Care and Emergency Medicine, University of Rwanda, Kigali, Rwanda; 2 Department of General Medicine, University of Rwanda, Kigali, Rwanda; 3 Department of Surgery, University of Minnesota, Minnesota, USA; 4 Department of Surgery, University Teaching Hospital of Kigali, Kigali, Rwanda; 5 Department of Internal Medicine, Kigali University Teaching Hospital, Kigali, Rwanda; 6 Department of Internal Medicine, Division of Infectious Diseases, University of Virginia, Charlottesville, USA; 7 Department of Anesthesiology, University of Virginia, Charlottesville, USA

**Keywords:** hand hygiene, hospitals, intensive care units, less-developed countries, Rwanda

## Abstract

**Background:**

Proper hand hygiene (HH) practices have been shown to reduce healthcare-acquired infections. Several potential challenges in low-income countries might limit the feasibility of effective HH, including preexisting knowledge gaps and staffing.

**Aim:**

We sought to evaluate the feasibility of the implementation of effective HH practice at a teaching hospital in Rwanda.

**Methods:**

We conducted a prospective quality improvement project in the intensive care unit (ICU) at the Kigali University Teaching Hospital. We collected data before and after an intervention focused on HH adherence as defined by the World Health Organization ‘5 Moments for Hand Hygiene’ and assuring availability of HH supplies. Pre-intervention data were collected throughout July 2019, and HH measures were implemented in August 2019. Post-implementation data were collected following a 3-month wash-in.

**Results:**

In total, 902 HH observations were performed to assess pre-intervention adherence and 903 observations post-intervention adherence. Overall, HH adherence increased from 25% (222 of 902 moments) before intervention to 75% (677 of 903 moments) after intervention (*P* < 0.001). Improvement was seen among all health professionals (nurses: 19–74%, residents: 23–74%, consultants: 29–76%).

**Conclusions:**

Effective HH measures are feasible in an ICU in a low-income country. Ensuring availability of supplies and training appears key to effective HH practices.

Intensive care units (ICUs) are magnets for drug-resistant organisms. Patients in the ICU are highly susceptible to healthcare-acquired infections (HAIs) due to severity of illness, frequent and intense healthcare worker contact, antimicrobial use, invasive medical devices, and close proximity to infected patients. HAIs are the leading cause for the emergence and spread of multidrug resistant pathogens. Broad spectrum antibiotics are used more frequently in ICUs than in other hospital departments. Thus, drug-resistant organisms are more likely to be present (1-3).

Epidemiological data from low- and middle-income countries (LMICs) show that antimicrobial resistance (AMR) is a substantial threat, with major economic and health consequences (4). Both the World Health Organization (WHO) when discussing the global status of AMR (5) and the ‘Review on Antimicrobial Resistance’ commissioned by the UK government (6) characterize AMR as an issue of global importance. A previous study done at the Kigali University Teaching Hospital (KUTH) ICU showed that sepsis was the most common diagnosis (34%). Organisms isolated most frequently included *Klebsiella* species and *Acinetobacter* species with high resistance levels to third-generation cephalosporins (7).

HAIs are more common in LMICs than in high-income countries (HICs). The incidence of HAIs in ICUs in LMIC is estimated to be at least three times higher (47.9 per 1,000 patient-days; 95% CI, 36.7–59.1) than that reported from the United States (8).

Hand hygiene (HH) measures as defined by WHO guidelines (9) are effective in reducing HAIs in HICs. Use of alcohol hand rub reduces HAIs by 36% (10, 11), and various studies indicate that HH measures are a cost-effective method of preventing HAIs (12, 13). However, it is far from certain that such HH interventions would be feasible and effective in LMICs. Many such settings lack an established culture of infection control with associated systems and funding. Hospital infection control committees may not focus attention on the ICU, and HH measures in critically ill patients often are not assessed or enforced. Hospital infection control efforts in low-income countries are hindered by a lack of awareness of the problem, lack of personnel, poor water supply, erratic electricity supply, ineffective antibiotic stewardship policies, and poor laboratory resources (14). Frequently, no identified champions are available to take a leadership role in the ICU. Many facilities in LMICs have limited human resources, low staff to patient ratios, and thus each staff member will be contacting many more patients than would be the case in a HIC ICU. This makes the need for scrupulous HH arguably more important in LMICs, while at the same time, understaffing increases time pressure so that HH measures may be skipped. Compounding these issues is a chronic and often profound shortage of even basic infection prevention materials such as alcohol gel and gloves. For all these reasons, it cannot be assumed that HH approaches that have been shown effective in HIC settings will be feasible in LMIC ICUs.

Substantial evidence from high-income settings demonstrates that HH measures can be implemented easily and are extremely effective. However, surprisingly few studies have evaluated the feasibility and efficacy of basic HH measures in LMICs, despite the major implications for approaching ICU infection prevention as a whole in LMICs. We hypothesized that HH practices can be effectively introduced with high compliance in an LMIC ICU. We investigated the feasibility of implementing an effective HH practice infection control training bundle and assuring availability of HH supplies and studied the effects on provider behavior at a representative low-income country (LIC) ICU at KUTH in Rwanda.

## Methods

We conducted a prospective observational study of the implementation of a HH bundle at the KUTH ICU. KUTH is a public tertiary referral and teaching hospital with approximately 540 beds located in the capital of Rwanda, Kigali. It has an emergency department, inpatient internal medicine, surgery, pediatrics, obstetrics and gynecology wards, and ICU. Its catchment area includes more than 19 district hospitals in Kigali and the adjacent Northern and Southern provinces.

The ICU has seven adult ICU beds and an adjacent high-dependency (step-down) unit with four adult beds. All 11 beds were studied in this project. The unit is staffed by a total of six anesthesiologists, five of whom were present during the implementation. One anesthesiologist staffs the ICU each week and is supported by two to three residents from both anesthesiology and surgery departments. A total of 33 nurses work in the ICU, six or seven of whom are present during the day shift, each caring for one to three patients. In addition, one nutritionist and two aides support the unit, and nursing and medical students rotate there.

On average, the ICU admits 25–35 patients per month with surgical (including trauma), neurosurgical, medical, and obstetrical conditions requiring intensive care. Occasionally, pediatric patients are admitted when the three-bed pediatric ICU is full.

The study was performed in three phases.

### Phase 1

#### Pre-intervention.

The aim was to target a large sample size of around 900 observations in pre- and post-interventions based on a literature review of similar studies (15-17). We collected pre-intervention data on HH adherence from July 18 to August 9, 2019 by using the WHO ‘My 5 Moments of Hand Hygiene’: before patient contact, before aseptic technique, after body fluid risks, after patient contact, and after contact with patient surroundings (18, 19). HH was primarily performed using a water basin and soap stationed in the ICU, in addition to supplies of alcohol gel distributed to each patient’s bed to mitigate frequent shortages of water. Dedicated research personnel were trained on how to use the WHO 5 Moments of Hand Hygiene observation tool (two sessions of 1 h each) and stationed in the ICU to observe and audit compliance with HH practices. Research personnel evaluated providers at each opportunity to interact with patients during at least three 8-h periods each week. Availability of hand washing supplies (water, soap, and alcohol) and their uses relating to WHO 5 moments were also assessed.

### Phase 2

#### Intervention.

We implemented proper HH according to WHO HH recommendations. In August 2019, we organized short, in-person training with two sessions (3 h each) for training on proper HH focused on the WHO 5 Moments. Infection prevention and control bundle courses in task-appropriate infection control measures were organized for all personnel with patient access in the ICU: physicians, nurses, aides, nutritionist, students at various levels, and support personnel. In order to optimize inter-auditor reproducibility, training sessions incorporated observation and scoring of standardized patient care episodes according to WHO guidelines, with feedback on accuracy and consistency. Cognitive aids in the form of posters and handouts were prepared and distributed. Availability of HH materials (alcohol gel, etc.) was ensured.

### Phase 3

#### Post-intervention.

After a 3-month ‘wash-in’ period, we collected post-intervention data from December 1, 2019 to January 9, 2020 using the same methodology as during the pre-intervention phase. Observers were trained for two sessions of 1 h each on how to use a WHO 5 Moments observation tool. Observers audited again as in Phase 1. The data collection form was created in Epi Info 7.0 (Centers for Disease Control and Prevention [CDC], Georgia, USA) using the WHO 5 Moments observation tool. The ICU health workers were aware of pre- and post-intervention data collection.

### Statistical Analysis

Data were collected and analyzed using Epi Info 7.0 (CDC) and Microsoft Excel (Microsoft Corporation, Washington, USA). We calculated descriptive statistics. Comparisons were made by Chi square test. *P* < 0.05 was considered statistically significant.

## Results

**Fig. 1.**
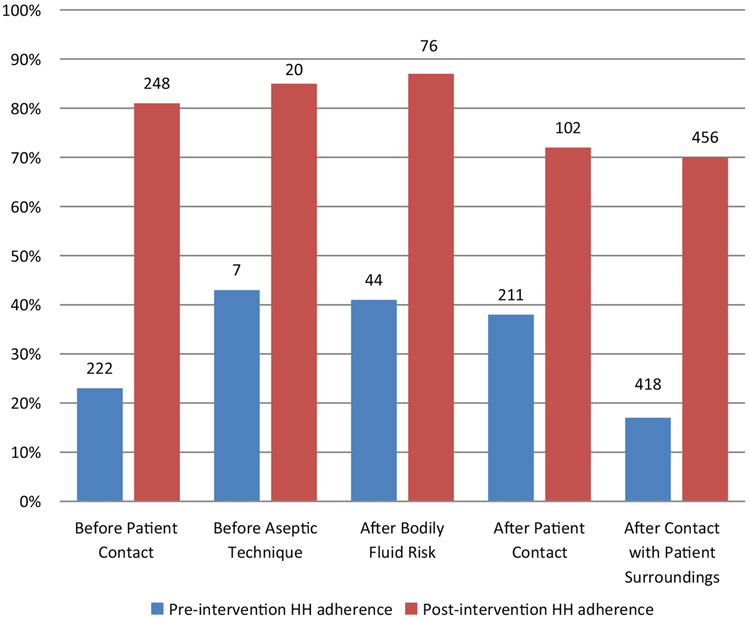
Adherence with World Health Organization’s ‘My 5 moments for Hand Hygiene’ before and after intervention. Number above bars indicates the number of observations for each group.

**Fig. 2.**
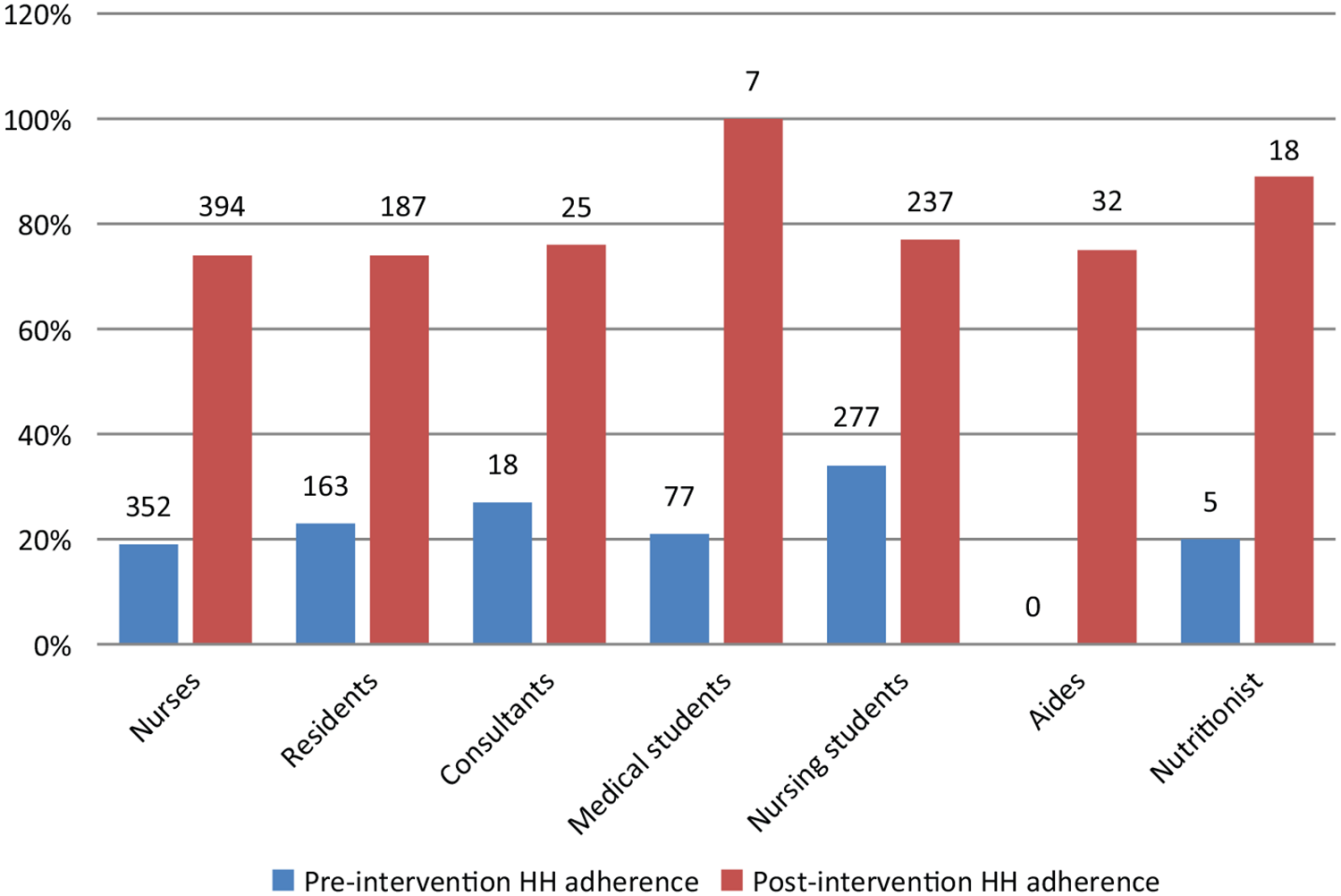
Adherence to hand hygiene protocols by staff group before and after intervention. Number above bars indicates the number of observations for each group.

In total, 902 HH observations were performed pre-intervention and 903 observations post-intervention. Overall, HH adherence tripled from 25% (222 of 902 moments) pre-intervention to 75% (677 of 903 moments) post-intervention (*P* < 0.001). Substantial improvements were noted for each of the WHO ‘5 Moments for Hand Hygiene’ opportunities (Fig. 1). The greatest improvement was noted before patient contact (absolute difference 58%); the smallest improvement was noted after patient contact (absolute difference 34%). All staff categories showed more than 70% compliance after intervention. Improvement was seen in each staff category (Fig. 2) with greatest improvement in medical students (absolute difference of 79%) and the smallest improvement in nursing students (absolute difference of 43%). Availability of alcohol-based hand wash increased from 76% to 99%.

## Discussion

Our results suggest that the implementation of effective HH measures in ICUs of LMICs is as feasible as it is in higher-resourced settings. Although not a focus of our study, other studies in HICs have associated the implementation of HH with reductions in HAIs (9), suggesting that the same might be the case in LMICs. The important factors were engagement of all personnel, focused training with reminders in the form of cognitive aids, and availability of HH materials, which include a regular supply of water, soap, gloves, and alcohol gel.

This ICU has two main water sinks for hand washing, but frequent water cut-offs occur. During the study period from pre-implementation to post-implementation, we ensured the presence of other HH materials like alcohol gel, gloves, paper towels, etc. We involved the hospital leadership to continue supplying these HH materials.

Although direct comparison of auditing practices is difficult, HH adherence in response to our education-based intervention appeared greater than in a similar multicenter collaborative study in the United States using product/volume usage measurement and feedback; in that study, the baseline ICU HH compliance increased from 26% to only 51% (20). In our setting, despite limited resources and low staff to patient ratio, HH compliance tripled.

Our pre-intervention findings were concordant with those of a systematic review, which found that baseline HH compliance is high in HICs at 64.5% but much lower in LICS (9–32%) (15, 21). The magnitude of improvement with an education-based HH program was near that of a study done at Swiss multisite regional hospital, where compliance increased from 61.4% to 83.4% after 18 months of the program (16). The large effect size seen in this study may be explained by a preexisting knowledge gap and lack of regular supplies of infection control materials, such as alcohol gel for hand cleaning and factors that are common in LMICs (22, 23). There remains a great need to emphasize infection prevention and control measures in LMIC nursing and medical schools (24, 25).

Although we did not investigate the impact of our intervention on HAIs specifically, other studies have shown that a decrease in HAI is a likely outcome of improved HH. A study in Vietnam in a low-resourced ICU studied the effect of proper HH adherence on HAI and cost of health care. HH adherence doubled from pre- to post-intervention (25.7–57.5%). This was associated with a reduction of HAI (from 31.7% to 20.3%) and substantial cost savings of $1,074 for each HAI prevented (26).

Studies have found that health workers are concerned about exposure to diseases after procedures. This may explain the high overall compliance after patient contact in our study (27). We found post-intervention HH adherence to be the highest in medical students and the nutritionist (only one nutritionist works at this unit). This finding contrasts with other studies, where compliance was higher among nurses (43%) than physicians (19%) and other health care workers (28%) (28, 29).

LMICs often are unable to invest in sufficient facilities for HH, thus creating a barrier for improving HH adherence. Therefore, for our implementation, we focused on basic, low-cost WHO-recommended steps (providing HH facilities, training, surveillance, and feedback) (30, 31).

Several limitations of our study should be recognized. Our ICU admits medical, surgical, and obstetrical patients, and as a result, residents and consultants from those departments make rounds in the unit. These personnel did not participate in the teaching and training but were audited during the pre- and post-intervention phases. This may have led to bias among health professional HH adherence, but if anything, it likely would have reduced any improvement noted. Another important consideration is that audits were performed only during regular day shifts, that is, Monday-Friday up to 7 pm. Further studies could monitor HH adherence during night shifts and weekends. We cannot rule out a study effect caused by the presence of audit personnel in the ICU. We tried to mitigate this as much as possible by waiting several months between training and post-intervention audits. Also, any study effect likely would have been similar during pre- and post-intervention periods, if not more pronounced during the pre-intervention period. Finally, we do not know if the effects observed will be sustained over longer time periods without repeat training.

We conclude from our findings that instituting effective HH measures is feasible and associated with high compliance in an ICU of an LIC. Our data suggest that regular supplies of infection control materials and training are key to effective HH practice. Further research is recommended to study any changes in HAI and multidrug resistant infections after these interventions.
